# Pulmonary hypertension in pregnancy: hemodynamic challenges, maternal risk, and multidisciplinary management

**DOI:** 10.1186/s44348-026-00096-3

**Published:** 2026-09-14

**Authors:** Sung-A Chang

**Affiliations:** 1 Division of Cardiology, Department of Medicine, Heart Vascular Stroke Institute, Samsung Medical Center, Sungkyunkwan University School of Medicine, Seoul, Republic of Korea; 2 Pulmonary Hypertension Center, Heart Vascular Stroke Institute, Samsung Medical Center, Seoul, Republic of Korea

**Keywords:** Pulmonary Hypertension, Pregnancy, Ventricular Function, Right, Postpartum Period, Echocardiography, Diagnostic Imaging, Patient Care Team

## Abstract

Pulmonary hypertension (PH) during pregnancy remains a high-risk clinical condition because normal gestational cardiovascular adaptation may exceed the reserve of the right ventricular (RV)-pulmonary circulation unit. Pregnancy is characterized by progressive increases in plasma volume, heart rate, stroke volume, and cardiac output, together with a reduction in systemic vascular resistance. These changes become particularly hazardous during labor, delivery, and the early postpartum period, when abrupt shifts in preload, venous return, and filling pressures may precipitate RV failure, hypoxemia, arrhythmia, or circulatory collapse. However, pregnancy-related risk in PH is heterogeneous and depends on the underlying PH phenotype, pulmonary vascular disease severity, RV function, oxygenation, and access to expert multidisciplinary care. This review summarizes the hemodynamic challenges of pregnancy in women with PH, phenotype-specific maternal and fetal risks, contemporary outcome data, treatment considerations, delivery planning, and postpartum management. Particular emphasis is placed on the first 24 to 72 h postpartum as a vulnerable period of hemodynamic stress and on the role of imaging, particularly echocardiographic surveillance, in identifying maladaptive RV responses and guiding timely escalation of care.

## Background

Pregnancy is a dynamic cardiovascular state characterized by progressive hemodynamic changes throughout gestation, with additional circulatory stress during delivery and the early postpartum period. Although these physiologic adaptations are generally accommodated in women with preserved cardiovascular reserve, they may unmask or exacerbate underlying cardiac disease and, in severe cases, lead to life-threatening maternal complications. Pulmonary hypertension (PH), regardless of etiology, is well recognized as a condition that increases maternal cardiovascular risk during pregnancy and after delivery, and pregnancy has therefore traditionally been discouraged in these patients [1–5]. In reality, however, clinicians continue to encounter pregnant women with PH, either because pregnancy occurs despite prior counselling or because the diagnosis is made only after conception [3, 4, 6, 7].

In recent years, outcomes have improved in selected patients with the introduction of multidisciplinary care, advances in targeted therapy in pulmonary arterial hypertension (PAH), and better intensive care unit (ICU) support [3, 6–10]. As a result, a simple categorical view of pregnancy as contraindicated is no longer sufficient. Greater emphasis should instead be placed on identifying risk heterogeneity and tailoring management according to the underlying phenotype and disease severity [1–4, 9, 11]. In this review, we discuss the hemodynamic challenges of pregnancy in women with PH, the spectrum of maternal and fetal complications, and contemporary approaches to risk stratification, monitoring, and multidisciplinary management.

## Hemodynamic adaptation during pregnancy and its consequences in PH

### Normal cardiovascular adaptation during pregnancy

During pregnancy, the maternal cardiovascular system undergoes broad hemodynamic adaptation to meet the metabolic demands of the fetus and placenta. These changes cannot be explained solely by expansion of blood volume; they are also mediated by increased estrogen and progesterone levels and by several vasoactive pathways. Estrogen contributes to endothelial function and increased nitric oxide bioavailability, thereby promoting vasodilation, while progesterone contributes to vascular smooth-muscle relaxation and a reduction in systemic vascular resistance (SVR). As a result, SVR begins to fall early in pregnancy, and blood pressure often decreases modestly until mid-gestation [5, 12].

The decrease in SVR induces compensatory cardiovascular changes. In the setting of lower peripheral resistance, heart rate and stroke volume increase to accommodate placental circulation and increased maternal metabolic demand. Cardiac output therefore rises substantially above the prepregnancy level. Plasma volume also increases progressively with advancing gestation, raising preload and contributing further to increased stroke volume and cardiac output. In women with normal cardiovascular function, these changes remain within the range of physiologic adaptation, and both the left ventricle (LV) and right ventricle (RV) can usually accommodate the increased flow demand [5, 12].

**Fig. 1:**
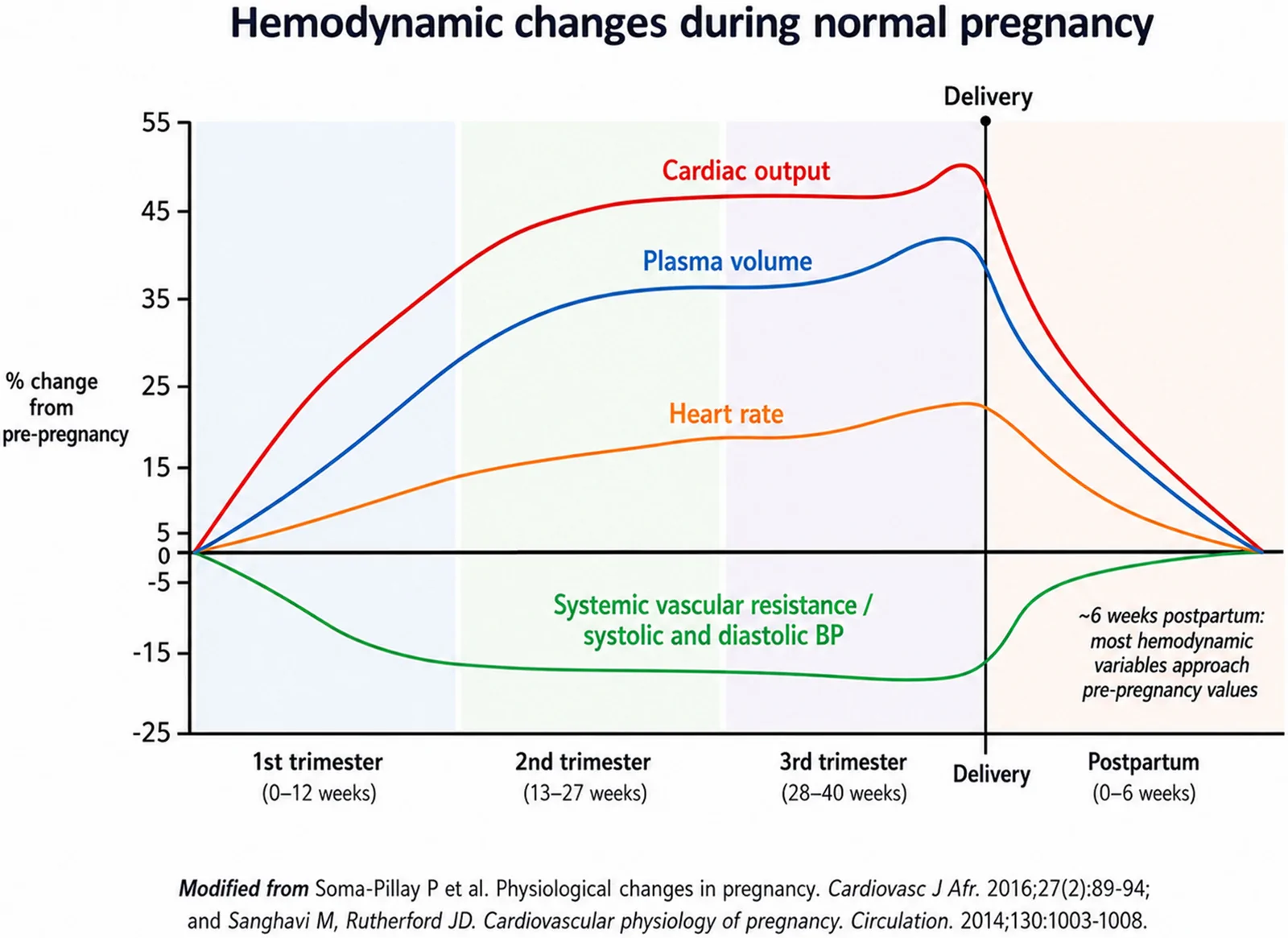
Hemodynamic changes during normal pregnancy. Cardiac output, plasma volume, and heart rate progressively increase during gestation, whereas systemic vascular resistance and blood pressure decrease. Most hemodynamic variables return toward prepregnancy levels by approximately 6 weeks postpartum. Data based on Soma-Pillay et al. [12] and Sanghavi and Rutherford [13]

These adaptations are not static throughout pregnancy. Cardiac output rises clearly by mid-gestation, while plasma volume and cardiac output approach their peak during the third trimester. During labor and delivery, uterine contractions, pain, sympathetic activation, possible hemorrhage, anesthesia-related changes in vascular tone, and shifts in central blood volume together produce greater hemodynamic variability. Immediately after delivery, autotransfusion from the contracting uterus and redistribution of extracellular fluid may abruptly increase preload. Thus, hormone-mediated vasodilation, blood volume expansion, and increases in heart rate and cardiac output are physiologic features of normal pregnancy, but they may become clinically important stressors in patients with limited cardiovascular reserve [5, 10, 12, 13]. The overall trajectory of these physiologic hemodynamic changes during normal pregnancy is summarized in Fig. 1 [12, 13].

### Why these changes are poorly tolerated in PH

In patients with PH, the hemodynamic adaptations normally required during pregnancy may be insufficiently accommodated and may instead impose excessive stress on the RV-pulmonary circulation unit. In normal pregnancy, SVR decreases and cardiac output increases, allowing the augmented flow to be distributed across the systemic and placental circulations. In PH, however, the increased cardiac output must traverse a structurally or functionally abnormal pulmonary vascular bed, and pulmonary vascular resistance (PVR) cannot fall to the degree expected in normal pregnancy [1, 2, 4, 5].

Consequently, the RV must cope simultaneously with increased flow demand and persistently elevated PVR. When PVR remains fixed or incompletely reversible, increased flow into the pulmonary circulation may further increase pulmonary artery pressure and aggravate RV pressure overload. Unlike the LV, the RV is normally adapted to a low-pressure pulmonary circulation and is therefore relatively vulnerable to abrupt or sustained pressure overload. Pregnancy-related increases in preload and cardiac-output demand may therefore lead to a rise in pulmonary artery pressure and RV afterload. Once this process exceeds RV compensatory capacity, RV dilatation and systolic dysfunction may develop [1, 2, 5, 10].

As RV function deteriorates, forward flow falls, tricuspid regurgitation (TR) may worsen, and right atrial (RA) pressure may rise. The dilated RV can also shift the interventricular septum leftward, impairing LV filling through ventricular interdependence. This mechanism reduces LV preload and systemic cardiac output, resulting in hypotension, syncope, end-organ hypoperfusion, and hypoxemia. The central problem in pregnant women with PH is therefore not simply an elevated pulmonary artery pressure, but a mismatch between gestational circulatory demand and the limited adaptive capacity of the RV-pulmonary circulation unit. This maladaptation forms the core mechanism linking pregnancy-related hemodynamic stress to clinical deterioration and maternal risk [1, 2, 10].

### Critical periods of clinical deterioration

Clinical deterioration in women with PH can occur at any time during pregnancy, but risk is concentrated during specific periods. From the late second trimester into the third trimester, progressive increases in plasma volume and cardiac output impose a growing burden on the RV. During this period, new or worsening exertional intolerance, dyspnea, edema, hypoxemia, or signs of right heart failure may emerge [1, 3, 4, 6, 10].

Labor and delivery represent another high-risk period. Uterine contractions, pain, sympathetic activation, blood loss, anesthesia-related changes in vascular resistance, and rapid shifts in preload and afterload occur simultaneously. In patients with limited RV reserve, these abrupt changes alone may precipitate low cardiac output, arrhythmia, hypotension, or right heart failure [1, 3, 5, 10].

The early postpartum period deserves particular attention. Immediately after delivery, autotransfusion from uterine contraction, mobilization of peripheral edema, and redistribution of extracellular fluid increase central blood volume and may acutely raise RV preload. A patient who appeared clinically stable during pregnancy or immediately after delivery may therefore develop sudden RV failure or circulatory instability in the early postpartum period. For this reason, delivery should not be interpreted as the end of risk in women with PH; close and intensive monitoring should continue after delivery, particularly in patients with PAH, severe PH, RV dysfunction, cyanosis, or recent clinical instability [1, 3, 4, 6, 10, 12, 13].

**Fig. 2:**
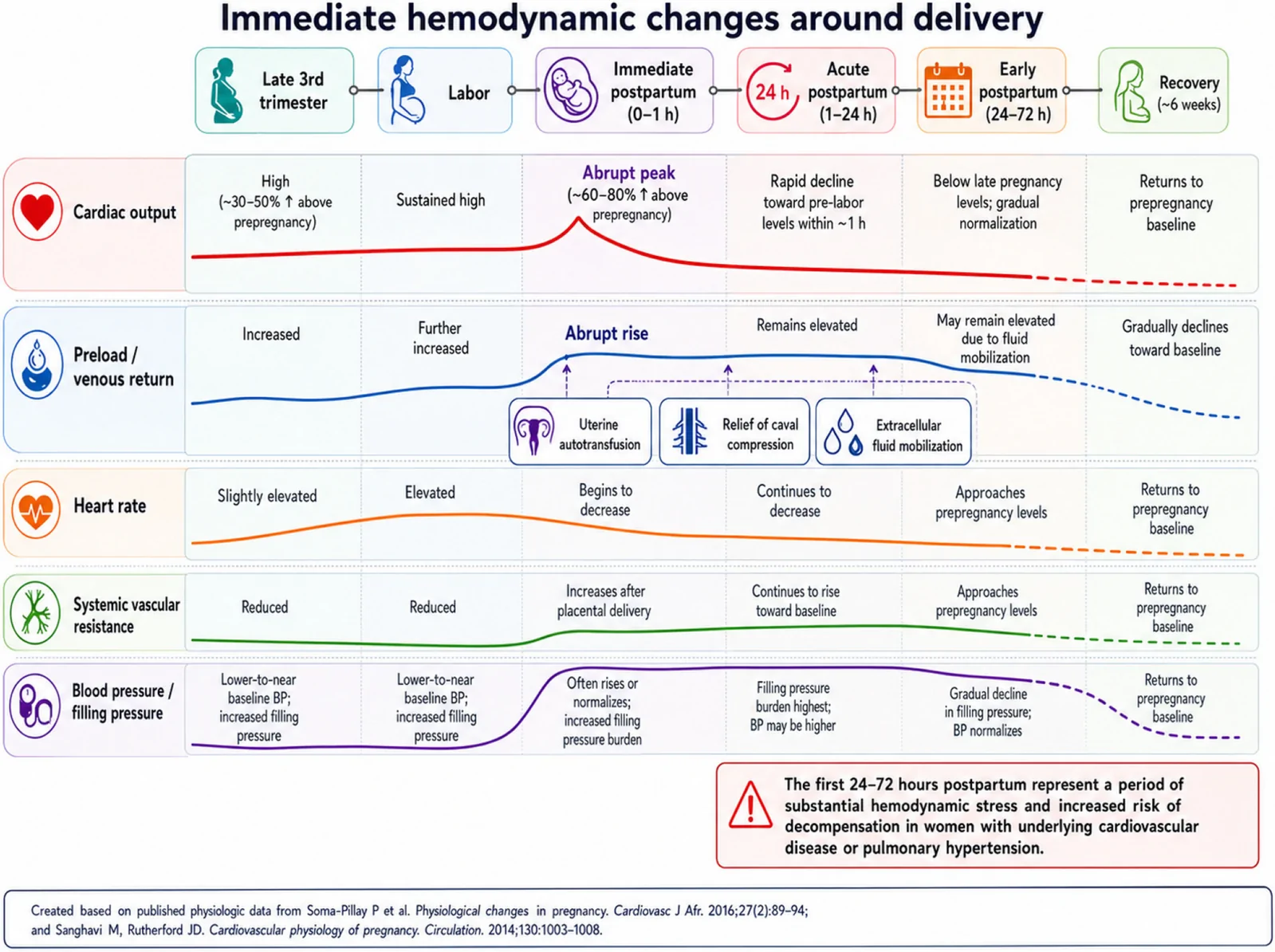
Immediate hemodynamic changes around delivery and early postpartum vulnerability in women with pulmonary hypertension. The figure summarizes the dynamic cardiovascular changes that occur from late pregnancy through labor, delivery, and the early postpartum period. Around delivery, uterine contractions, uterine autotransfusion after placental delivery, relief of caval compression, and mobilization of extravascular fluid can abruptly increase venous return and cardiac output. Although cardiac output may decline rapidly toward prelabor levels within the first hour after delivery, increased preload and filling pressure burden may persist during the first 24 to 72 h postpartum. In women with pulmonary hypertension, these abrupt changes may exceed the adaptive capacity of the right ventricular–pulmonary circulation unit, leading to right ventricular dilation or dysfunction, impaired left ventricular filling, systemic hypoperfusion, hypoxemia, arrhythmia, or right heart failure. Based on published physiologic data from Soma-Pillay et al. [12] and Sanghavi and Rutherford [13]

Figure 2 illustrates the immediate hemodynamic changes around delivery and the early postpartum period [12, 13]. The schematic emphasizes that uterine autotransfusion, relief of caval compression, and extracellular fluid mobilization can abruptly increase venous return and filling pressure burden after delivery. In women with PH, these changes may exceed the adaptive capacity of the RV-pulmonary circulation unit, leading to RV dilation or dysfunction, septal shift, impaired LV filling, and reduced systemic output [1, 2, 5, 10, 12, 13].

## Risk heterogeneity according to PH etiology

Although pregnancy in PH is often discussed as a uniformly high-risk condition, the magnitude and mechanism of risk vary substantially according to the underlying PH phenotype. Current guidelines and expert consensus statements continue to regard PAH and severe PH as conditions associated with prohibitive maternal risk, yet contemporary data also suggest that pregnancy outcome is influenced not only by the diagnostic label, but also by the severity of pulmonary vascular disease, RV functional reserve, oxygenation status, and the availability of expert multidisciplinary care [1–4, 6–9, 11].

Group 1 PAH is regarded as a high-risk condition during pregnancy, irrespective of its specific subtype. In idiopathic, heritable, connective tissue disease-associated, and congenital heart disease-associated PAH, pregnancy imposes additional circulatory demand on a pulmonary vascular bed with limited vasodilatory capacity. As plasma volume and cardiac output increase, the RV must accommodate greater flow across a fixed or only partially reversible elevation in PVR. Patients with preserved RV function, low-risk clinical profile, and well-controlled hemodynamics may have better outcomes in selected series, but these favorable observations should not be generalized to all PAH patients [6–9]. In particular, connective tissue disease-associated PAH may carry additional risk because systemic inflammation, renal involvement, interstitial lung disease, thrombosis, or concomitant left heart involvement can further reduce cardiopulmonary reserve.

Among PAH phenotypes, Eisenmenger syndrome remains one of the most dangerous forms during pregnancy. Its risk is not explained solely by elevated pulmonary artery pressure. Rather, the combination of fixed high PVR, right-to-left or bidirectional shunting, cyanosis, and limited ability to increase pulmonary blood flow creates extreme hemodynamic fragility [1, 14, 15]. The physiological fall in SVR during pregnancy may aggravate right-to-left shunting, leading to worsening maternal hypoxemia and reduced systemic oxygen delivery. This mechanism also provides a direct link between maternal and fetal risk. Maternal hypoxemia compromises fetoplacental oxygen delivery and is associated with preterm delivery, low birth weight, and fetal loss. Therefore, Eisenmenger physiology should be viewed as a dual-risk phenotype, in which the maternal RV-pulmonary circulation unit and fetoplacental oxygen delivery are simultaneously compromised [14, 15].

PH due to left heart disease has a different pathophysiological basis, but it is not necessarily benign. In isolated postcapillary PH, the primary stressor is the rise in left-sided filling pressure during pregnancy-related volume expansion and tachycardia. Pulmonary congestion, atrial arrhythmia, and heart failure may become dominant clinical problems. In contrast, combined postcapillary and precapillary PH may behave more like a pulmonary vascular disease phenotype, particularly when pulmonary vascular remodeling and RV dysfunction are present [2, 16, 17]. This distinction is clinically important when interpreting PH associated with mitral or aortic valve disease, cardiomyopathy, or diastolic dysfunction. PH accompanying valvular regurgitation or left heart disease should not automatically be equated with idiopathic PAH; however, when it is accompanied by significant RV dysfunction, high PVR, or recurrent heart failure, pregnancy risk may still be substantial [16–18].

Data on pregnancy in group 3 PH and group 4 PH are more limited than for PAH and left heart disease-associated PH. Pregnancy-specific evidence in group 3 PH is particularly sparse. Data from pregnant women with interstitial lung disease, although not specific to PH, indicate that more severe lung disease is associated with increased maternal and neonatal risk; in patients who also have PH, reduced gas exchange reserve and hypoxemia may further amplify maternal desaturation and fetal compromise [19]. In group 4 PH, chronic thromboembolic obstruction imposes a fixed mechanical component of RV afterload, while pregnancy and the postpartum period add a prothrombotic milieu and introduce anticoagulation complexity. Case reports suggest that pregnancy may be possible after successful pulmonary endarterectomy or balloon pulmonary angioplasty in carefully selected and closely monitored patients, but evidence remains too sparse to define this as a generally safe phenotype [20, 21].

Taken together, etiology-specific risk assessment should move beyond the binary presence or absence of PH. In practice, the most informative risk markers are severe pulmonary vascular disease, RV dysfunction, hypoxemia, cyanosis or shunt physiology, prior clinical instability, need for advanced therapy, and limited capacity to tolerate volume shifts. Thus, pregnancy-related risk in PH should be understood as the interaction between PH phenotype, pulmonary vascular burden, oxygenation, and RV adaptation rather than as a single uniform diagnosis. The major phenotype-specific risks are summarized in Table 1 [1–11, 14–21], and crosscutting modifiers that should refine pregnancy risk assessment are summarized in Table 2 [1–4, 6, 9–11, 14, 15, 19–21].

**Table 1:** Diagnostic category-based pregnancy risk in PH

Category	Practical pregnancy risk	Dominant mechanism	Key reference
**WHO group 1: PAH**			
Idiopathic, heritable, drug- or toxin-associated PAH	Very high; generally contraindicated	Fixed precapillary pulmonary vascular disease limits the normal pregnancy-related fall in PVR. Increased cardiac output and peripartum volume shifts may precipitate RV failure, PAH crisis, shock, or death	[1–4, 6–10]
Connective tissue disease-associated PAH	Very high; generally contraindicated	PAH-related RV afterload is compounded by systemic disease burden such as renal involvement, ILD, anemia, inflammation, thrombosis, or left heart involvement	[1–4, 6, 9, 11]
Congenital heart disease–associated PAH without Eisenmenger physiology	High to very high; individualized	Risk depends on residual anatomy, prior repair, shunt direction, PVR, cyanosis, RV adaptation, arrhythmia burden, and ventricular function. Monitor oxygen saturation, shunt physiology, RV function, pulmonary pressure trend, arrhythmia, and fetal growth	[1–4, 6–8]
Eisenmenger syndrome/cyanotic PAH-CHD	Extreme; prohibitive risk	Fixed high PVR with right-to-left or bidirectional shunting, cyanosis, paradoxical embolic risk, and inability to augment pulmonary flow make this one of the highest risk phenotypes. Maternal hypoxemia directly threatens fetal oxygen delivery	[1, 3, 9, 14, 15]
Portopulmonary PAH and other associated PAH	Very high; individualized by PH and systemic severity	The PAH component carries the usual RV afterload risk, while portal hypertension or systemic disease may add high-output physiology, coagulopathy, bleeding risk, hepatic dysfunction, infection risk, or drug interaction concerns	[1–4, 6, 9, 11]
**WHO group 2: PH associated with left heart disease**			
Valvular left heart disease (mitral/aortic disease; congenital or acquired)	Variable; high when PH, LV dysfunction, severe stenosis, or multivalve disease is present	Plasma volume expansion, tachycardia, and labor-related autotransfusion increase LA and pulmonary venous pressure. Secondary PH may worsen pulmonary edema risk and impose additional RV afterload	[1, 2, 16–18]
Cardiomyopathy, HFpEF/HFrEF, LV or LA disease	High to very high if symptomatic, EF impaired, or filling pressure elevated	Limited ability to accommodate preload and heart rate changes can increase PCWP, pulmonary venous hypertension, pulmonary edema, and secondary RV dysfunction	[1, 2, 16, 17]
Combined postcapillary and precapillary PH phenotype	High; closer to advanced cardiopulmonary risk than isolated postcapillary PH	Postcapillary congestion coexists with pulmonary vascular remodeling and elevated PVR; the RV faces both pulmonary venous hypertension and true afterload stress	[1, 2, 16, 17]
**WHO group 3: PH associated with lung disease and/or hypoxia**			
Interstitial lung disease or parenchymal lung disease–associated PH	High to very high when hypoxemia, reduced DLCO, or RV dysfunction is present	Precapillary PH is coupled to impaired gas exchange. Pregnancy may reduce respiratory reserve, and hypoxemia can intensify pulmonary vasoconstriction and RV afterload	[1, 2, 9, 11, 19]
COPD, sleep-disordered breathing, hypoventilation, or chronic hypoxic lung disease	Variable; high when significant hypoxemia, hypercapnia, or RV dysfunction is present	Hypoxic pulmonary vasoconstriction and limited ventilatory reserve may worsen during pregnancy, especially with infection, anemia, sleep-related hypoxemia, or peripartum respiratory stress	[1, 2, 5, 9, 11]
**WHO group 4: PH associated with PA obstructions**			
Untreated, inoperable, or residual CTEPH/CTEPD with PH	Very high when residual PH or RV dysfunction is present	Mechanical obstruction and secondary microvasculopathy increase PVR and RV afterload. Pregnancy adds hypercoagulability, anticoagulation complexity, and risk of recurrent thrombosis or acute PE superimposed on chronic obstruction	[1, 2, 9, 11, 20, 21]
Previously treated CTEPH after PEA or BPA with normalized/near-normal hemodynamics	Potentially lower, but evidence remains sparse; residual PH determines risk	Successful reduction of pulmonary vascular obstruction may improve pregnancy tolerance, but residual PH, impaired RV reserve, or recurrent thrombosis can still carry major risk	[1, 2, 20, 21]
**WHO group 5: PH with unclear and/or multifactorial mechanisms**			
Hematologic disorders (e.g., sickle cell disease, chronic hemolytic anemia, myeloproliferative disease)	High; individualized	Multiple mechanisms may coexist: anemia or high-output physiology, hemolysis-associated pulmonary vasculopathy, thrombosis, hypoxemia, renal disease, and systemic inflammation	[1, 2, 9, 11]
Systemic, metabolic, renal, inflammatory, or granulomatous disease–associated PH	Variable; high when RV dysfunction, hypoxemia, renal failure, or lung involvement is present	Mechanisms may include pulmonary vascular disease, left heart involvement, chronic hypoxia, volume overload, thromboembolism, or extrinsic vascular involvement	[1, 2, 9, 11]
Segmental or mechanical pulmonary vascular obstruction, fibrosing mediastinitis, tumoral obstruction, or other rare multifactorial causes	Uncertain but potentially high when obstruction or RV dysfunction is significant	Risk depends on the degree of vascular obstruction, hypoxemia, RV afterload, and feasibility of treating the underlying cause	[1, 2, 9, 11]

Risk levels are qualitative and intended for manuscript framing. They should be interpreted together with formal pregnancy risk assessment, hemodynamic severity, RV function, oxygenation, and expert cardio-obstetric/PH team evaluation

BPA, balloon pulmonary angioplasty; CHD, congenital heart disease; COPD, chronic obstructive pulmonary disease; CTEPD, chronic thromboembolic pulmonary disease; CTEPH, chronic thromboembolic pulmonary hypertension; DLCO, diffusing capacity of the lung for carbon monoxide; EF, ejection fraction; HFpEF, heart failure with preserved ejection fraction; HFrEF, heart failure with reduced ejection fraction; ILD, interstitial lung disease; LA, left atrial; LV, left ventricular; PA, pulmonary artery; PAH, pulmonary arterial hypertension; PCWP, pulmonary capillary wedge pressure; PEA, pulmonary endarterectomy; PE, pulmonary embolism; PH, pulmonary hypertension; PVR, pulmonary vascular resistance; RV, right ventricular; WHO, World Health Organization

**Table 2:** Crosscutting modifiers of pregnancy risk in PH

Modifier	Clinical implication in pregnancy
RV dysfunction, severe RV dilatation, rising BNP/NT-proBNP, syncope, or prior right heart failure	These findings identify limited RV reserve and increase the likelihood that normal gestational increases in cardiac output, delivery-related autotransfusion, or early postpartum volume shifts will trigger decompensation [1–4, 6, 9, 10]
Severe PH or high PVR, regardless of WHO group	Contemporary systematic reviews and cohort data suggest that disease severity may be more prognostically relevant than etiology alone [9, 11]
Cyanosis, resting/exertional hypoxemia, or oxygen dependence	Maternal hypoxemia affects both maternal pulmonary vasoconstriction/RV afterload and fetal oxygen delivery; fetal growth restriction and prematurity become key concerns [14, 15, 19]
Need for anticoagulation, CTEPH/CTEPD, thrombophilia, or prior venous thromboembolism	Pregnancy and the postpartum period are hypercoagulable; management must balance recurrent thrombosis/PE risk against delivery-related and hemorrhagic risk [1, 2, 20, 21]
Systemic disease burden (renal, hepatic, inflammatory, hematologic, or pulmonary parenchymal disease)	Noncardiac disease can independently determine maternal and fetal outcomes and may alter medication choice, volume management, and timing or mode of delivery [1, 2, 9, 11, 19]

BNP, B-type natriuretic peptide; CTEPD, chronic thromboembolic pulmonary disease; CTEPH, chronic thromboembolic pulmonary hypertension; NT-proBNP, N-terminal pro–B-type natriuretic peptide; PE, pulmonary embolism; PH, pulmonary hypertension; PVR, pulmonary vascular resistance; RV, right ventricular; WHO, World Health Organization

## Imaging assessment and surveillance during pregnancy

**Fig. 3:**
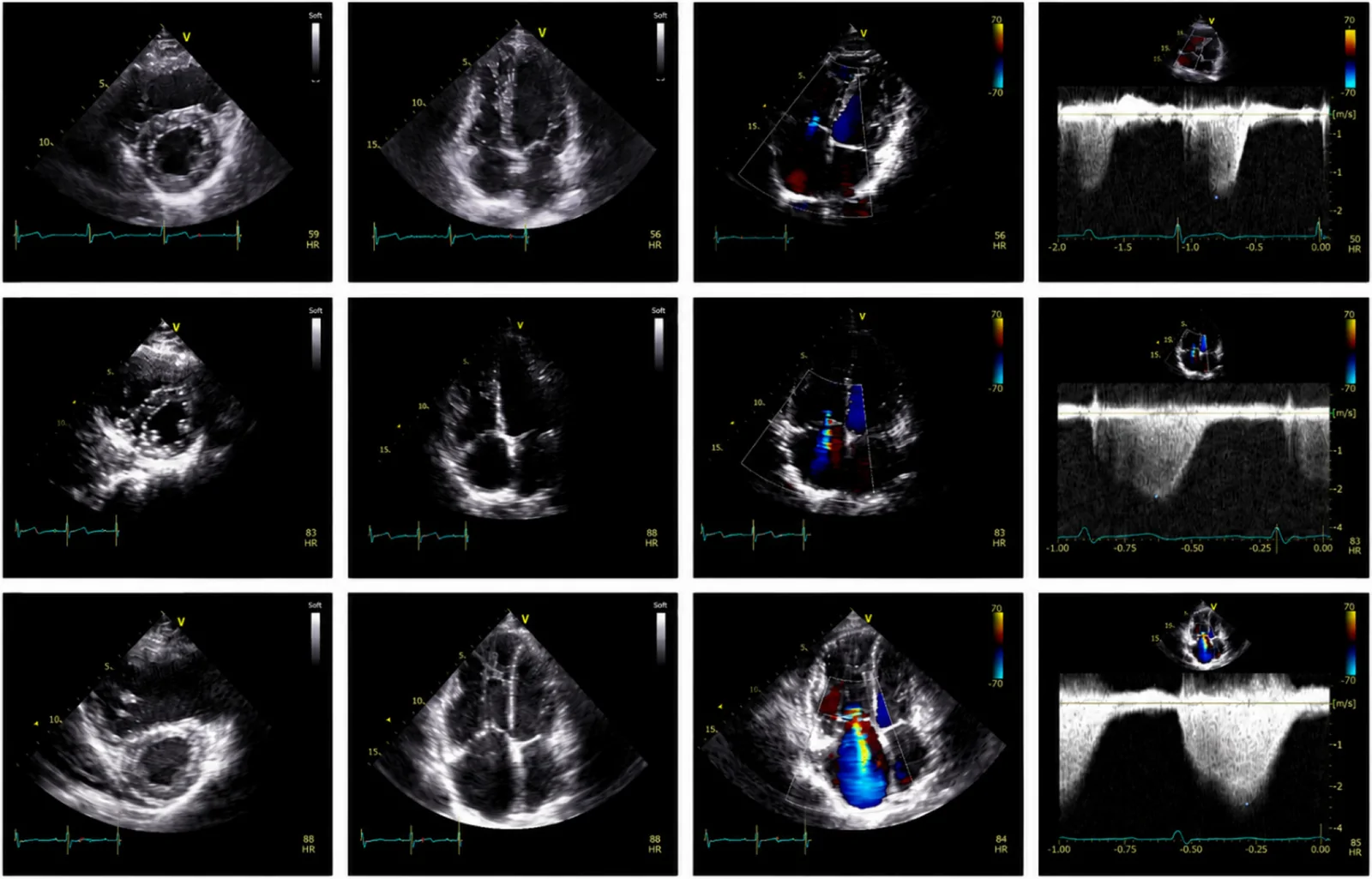
Longitudinal echocardiographic assessment of worsening pulmonary hypertension during pregnancy. Serial echocardiographic images demonstrate progressive right-sided cardiac remodeling and hemodynamic deterioration during pregnancy. This case highlights the value of serial echocardiography in monitoring right ventricular adaptation, identifying clinical deterioration, and supporting peripartum management in pregnant women with pulmonary hypertension. Panels correspond to the prepregnancy period (upper), 20 weeks of gestation (middle), and 33 weeks of gestation (bottom), respectively

In pregnant women with PH, imaging should be directed toward assessment of RV adaptation, early detection of hemodynamic deterioration, and guidance of peripartum monitoring intensity. Echocardiography is the first-line modality because it is noninvasive, repeatable, bedside-accessible, and does not involve ionizing radiation. Serial echocardiography can assess RV size and systolic function, RA enlargement, TR velocity, septal flattening, right-sided filling pressure surrogates, pericardial effusion, and impaired LV filling related to ventricular interdependence. In this setting, temporal change may be more informative than any single estimated pulmonary pressure value [1, 2]. A representative serial echocardiographic example of worsening PH during pregnancy is shown in Fig. 3.

Other imaging modalities should be used selectively according to the clinical question. Chest radiography may be useful for assessing cardiomegaly, pulmonary congestion, pleural effusion, or alternative pulmonary causes of dyspnea, particularly during the early postpartum period. Cardiovascular magnetic resonance can provide robust assessment of RV volumes and function when echocardiographic windows are limited, but gadolinium contrast should generally be avoided during pregnancy unless essential [1, 22]. Computed tomography pulmonary angiography or ventilation-perfusion imaging is not part of routine PH surveillance; however, these modalities should not be withheld when acute pulmonary embolism or clinically important thromboembolic disease is suspected and the result would alter management, because clinically indicated diagnostic imaging in pregnancy is supported when maternal benefit outweighs fetal radiation considerations [22].

## Monitoring during pregnancy and the postpartum period

There is no universally validated, gestational week–specific surveillance schedule for pregnant women with PH, and the intensity of monitoring should be individualized according to PH phenotype, RV function, oxygenation, functional class, natriuretic peptide trajectory, and obstetric status. Nevertheless, several guideline and consensus principles are consistent. The 2025 European Society of Cardiology (ESC) pregnancy guideline emphasizes management by a pregnancy heart team that includes a PH expert, close monitoring when pregnancy is continued, regular echocardiography and blood testing including natriuretic peptide levels when appropriate, and right heart catheterization (RHC) when diagnostic uncertainty or major therapeutic decisions require invasive clarification [1]. For Eisenmenger syndrome, the same guideline notes that regular follow-up is advisable initially every 2 to 4 weeks and then weekly in the third trimester, with careful assessment for worsening hypoxemia and heart failure symptoms [1]. The 2022 ESC/European Respiratory Society (ERS) PH guideline also provides the core PAH follow-up domains that can be adapted to pregnancy: clinical assessment including World Health Organization functional class, 6-min walk distance (6MWD), B-type natriuretic peptide (BNP) or N-terminal pro-BNP (NT-proBNP), electrocardiography, echocardiography or cardiac magnetic resonance imaging, oxygenation assessment, and RHC when clinically indicated [2]. The Pulmonary Vascular Research Institute statement similarly recommends close multidisciplinary monitoring, including monthly follow-up in the first and second trimesters and weekly visits in the third trimester, with clinical evaluation, echocardiography, and laboratory testing directed toward early detection of deterioration [4].

The timing of surveillance should reflect the non-linear hemodynamic stress imposed by pregnancy. Although some patients remain clinically stable during early gestation, clinical deterioration in PAH has been reported most frequently around 20 to 24 weeks, in the early third trimester, during delivery, and in the postpartum period, corresponding to phases of increasing cardiac output, plasma volume expansion, abrupt preload shifts, and heightened vulnerability to RV failure [4]. This temporal pattern supports a structured reassessment around mid-gestation, intensification after approximately 28 weeks, and particularly vigilant monitoring during the peripartum and early postpartum phases. In practice, an unexplained rise in NT-proBNP or BNP, decline in 6MWD, worsening oxygen saturation by pulse oximetry (SpO_2_), new congestion, syncope, increasing TR, RV enlargement or dysfunction, or fetal growth restriction should prompt earlier echocardiography and escalation of pregnancy heart team review [1, 2, 4].

Natriuretic peptide testing is useful because normal pregnancy does not usually produce marked elevations in BNP or NT-proBNP, whereas persistent or rising levels may indicate inadequate cardiovascular adaptation. In women with cardiovascular disease, serial biomarker assessment has been shown to be clinically informative during pregnancy, and an NT-proBNP threshold of approximately 200 pg/mL has been proposed for diagnosing heart failure or preeclampsia in pregnant cardiac patients [23]. In women with congenital heart disease, NT-proBNP measured around 20 weeks of gestation independently predicted cardiovascular events, supporting mid-gestation biomarker reassessment as a practical component of surveillance [24]. These data should not be interpreted as PH-specific cutoffs; rather, serial trends should be integrated with symptoms, oxygenation, RV function, and fetal status.

## Treatment during pregnancy

Treatment during pregnancy should aim to preserve RV reserve, maintain adequate systemic and uteroplacental oxygen delivery, and prevent abrupt changes in preload, afterload, oxygenation, and systemic blood pressure. Supportive care is therefore central. Furosemide may be used when right-sided congestion or volume overload develops, but the target should be euvolemia rather than dehydration because excessive preload reduction can lower cardiac output and systemic perfusion. Maternal hypoxemia should be corrected with supplemental oxygen, particularly when SpO_2_ is below 90% or PaO_2_ is below 60 mmHg, with pragmatic targets of SpO_2_ above 92% and PaO_2_ above 70 mmHg when feasible. Anemia and iron deficiency should be monitored closely and corrected, because reduced oxygen-carrying capacity may be poorly tolerated in PH. Patients should also receive dietary counselling to avoid excessive sodium and rapid fluid loading, especially in the early postpartum period; in Korea, this may require specific predelivery education regarding high-sodium traditional postpartum foods such as seaweed soup, while maintaining adequate nutrition [4, 5, 25, 26].

PAH-targeted therapy requires immediate review of fetal safety and maternal risk. Endothelin receptor antagonists, riociguat, and selexipag are not recommended during pregnancy and should be discontinued before conception whenever possible. If pregnancy occurs during exposure, the patient should be urgently referred to an expert PH pregnancy team for counselling and transition to pregnancy-compatible therapy. Among PAH-specific drugs, sildenafil and prostacyclin-based therapies have the greatest pregnancy experience. Sildenafil is the most commonly used oral option, whereas continuous parenteral prostacyclin, especially intravenous epoprostenol or treprostinil, should be considered in high-risk patients, progressive symptoms, RV dysfunction, or clinical deterioration. Inhaled prostacyclin or inhaled nitric oxide may be useful as adjunctive therapy in selected acute or peridelivery settings, but escalation to parenteral prostacyclin should not be delayed when RV failure progresses [1, 4, 25–27]. Table 3 summarizes the pregnancy-related use and practical considerations of PAH-targeted and PH-directed therapies.

**Table 3:** PAH-targeted and PH-directed therapies during pregnancy

Therapy	Use during pregnancy	Practical considerations
ERA (bosentan, ambrisentan, macitentan)	Contraindicated; not recommended	Stop before conception whenever possible. If exposure occurs, provide urgent expert counselling and transition to pregnancy-compatible therapy
Riociguat	Contraindicated; not recommended	Avoid during pregnancy because of fetal safety concerns; consider alternative PAH therapy (PDE5I)
Selexipag	Not recommended	Human pregnancy data are insufficient, and 2025 ESC pregnancy guidance lists selexipag among drugs not recommended during pregnancy
PDE5 inhibitors (sildenafil, tadalafil)	May be used when PAH therapy is required	Sildenafil has the largest pregnancy experience and is generally preferred. Data for tadalafil are more limited; continuation or switching should be individualized
IV epoprostenol	Preferred escalation option for high-risk PAH or RV deterioration	Most established prostacyclin experience in pregnancy. Consider early initiation in severe disease, WHO-FC III–IV symptoms, RV dysfunction, or worsening biomarkers/oxygenation
Treprostinil (SC/IV; inhaled in selected cases)	May be considered when clinically indicated	Useful when parenteral prostacyclin is needed or already established
Inhaled iloprost/inhaled prostacyclin	Adjunctive option in selected patients	May reduce pulmonary vascular tone with less systemic hypotension, but short duration and rebound risk limit use as sole therapy in unstable disease
Calcium channel blockers	Only for true vasoreactive PAH responders	Do not use empirically in nonvasoreactive PAH. Continue only when acute vasoreactivity and sustained clinical benefit are documented
Inhaled nitric oxide	Acute or peridelivery adjunct, not chronic maintenance therapy	May improve oxygenation and pulmonary vascular tone with limited systemic hypotension. Use in monitored expert-center settings as part of an escalation plan

ERA, endothelin receptor antagonist; ESC, European Society of Cardiology; IV, intravenous; PAH, pulmonary arterial hypertension; PDE5, phosphodiesterase type 5; PDE5I, phosphodiesterase type 5 inhibitor; PH, pulmonary hypertension; RV, right ventricle; SC, subcutaneous; WHO-FC, World Health Organization functional class

## Delivery planning and peripartum/postpartum care

The timing of delivery should be individualized and should first take into account standard obstetric and fetal indications. When severe fetal growth restriction, fetal distress, preterm labor, placental complications, or other obstetric emergencies are present, the timing and mode of delivery should follow obstetric indications [28]. In the absence of such indications, delivery planning in high-risk women with PAH or severe PH should be guided primarily by maternal cardiopulmonary risk. Although randomized data are lacking and current guidelines do not define a single optimal gestational age, expert consensus and contemporary reviews support planned delivery within approximately 34 to 36 weeks in clinically stable high-risk patients, with earlier delivery considered when symptomatic decline, worsening RV function, progressive hypoxemia, rising biomarkers, or hemodynamic instability occurs [1, 4–6, 29, 30]. In our institutional practice, planned delivery around 35 weeks is often considered in selected high-risk patients without competing obstetric indications, because continuation to full term may expose the mother to further increases in plasma volume, venous return, and RV load. This maternal risk strategy must be balanced against neonatal risks related to late-preterm birth; however, population-based neonatal data suggest that although morbidity is increased at 35 to 36 weeks compared with term birth, the absolute risk of severe neonatal morbidity remains relatively low in contemporary care settings [31]. Thus, planned delivery around 35 weeks should not be interpreted as a universal gestational age rule, but rather as a risk-balanced approach that integrates maternal RV reserve, oxygenation, PAH control, fetal growth and maturity, and neonatal ICU availability. Delivery should be planned in advance by a multidisciplinary team that includes maternal–fetal medicine, cardiology or PH specialists, anesthesiology, ICU medicine, neonatology, and pediatric specialists. Before delivery, this team should review the maternal risk profile, fetal status, anesthesia plan, monitoring strategy, PAH medication plan, fluid and vasopressor strategy, anticipated need for ICU care, and neonatal support. The mode of delivery should be determined primarily by the obstetric team in collaboration with the multidisciplinary team, rather than by PH diagnosis alone, because robust evidence supporting routine vaginal delivery or routine cesarean delivery for all PAH patients is lacking [1, 5, 30].

**Fig. 4:**
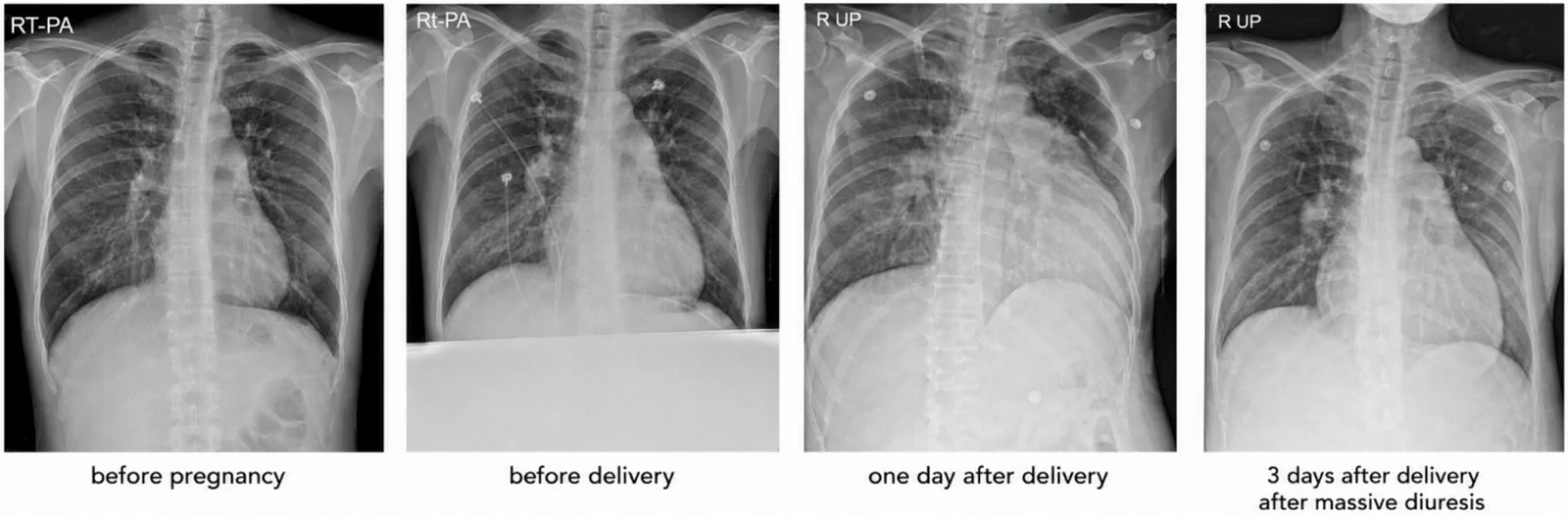
Transient cardiomegaly during the early postpartum period in women with pulmonary arterial hypertension. Serial chest radiographs from two representative patients demonstrate abrupt enlargement of the cardiac silhouette on postpartum day 1, followed by improvement by postpartum day 3, highlighting the dynamic hemodynamic burden of the immediate postpartum period

The immediate postpartum period requires the same level of anticipatory planning. After delivery, uterine contraction, relief of caval compression, and mobilization of extravascular fluid collectively increase venous return and impose an abrupt preload challenge on the pressure-overloaded RV. Contemporary systematic data indicate that a substantial proportion of maternal deaths in PAH occur during the first several days after delivery, with 61% reported at 0 to 4 days postpartum, while critical care reviews emphasize the first 48 h and the first postpartum week as particularly vulnerable periods [6, 10]. Therefore, postpartum management should focus on meticulous rather than indiscriminate volume reduction. Volume overload, pulmonary congestion, rising right-sided filling pressures, or increasing NT-proBNP should prompt active diuretic therapy and strict fluid balance, whereas excessive preload depletion should be avoided because the failing RV remains preload-sensitive [1, 10]. In high-risk patients, care should be continued in an ICU or equivalent monitored setting, with continuous electrocardiographic and SpO_2_ monitoring, careful blood pressure support, close assessment of urine output, renal function, and electrolytes, and repeated evaluation of RV function when clinical status changes [1, 10]. After delivery, PAH-targeted therapy should be reassessed promptly. Drugs avoided during pregnancy because of fetal risk, particularly endothelin receptor antagonists, may be reintroduced postpartum when clinically indicated, once bleeding risk, hepatic function, drug interactions, and overall maternal stability have been reviewed [1, 32]. In women with severe PAH, postpartum deterioration, or insufficient control on pregnancy-compatible therapy alone, escalation of PAH treatment should not be delayed solely to preserve lactation. Breastfeeding may be discussed on an individualized basis in clinically stable women receiving drugs for which very limited drug-specific human milk exposure data are available, but the evidence base remains sparse [33]. When maternal stabilization requires therapy with insufficient lactation safety data, avoidance or discontinuation of breastfeeding may be appropriate after multidisciplinary counselling. In this setting, the primary therapeutic priority should be maternal survival, RV recovery, and durable PAH control, with lactation decisions made through shared counselling involving the PH team, obstetric team, neonatology, and the patient [1, 32, 33]. Representative serial chest radiographs demonstrating transient early postpartum cardiomegaly and recovery are shown in Fig. 4.

## Conclusions

Pregnancy in women with PH remains a high-risk condition, particularly in PAH, Eisenmenger physiology, severe precapillary PH, hypoxemic lung disease, and any phenotype accompanied by impaired RV reserve. Nevertheless, contemporary experience suggests that outcomes have improved in selected patients as a result of PAH-targeted therapy, structured surveillance, planned delivery in expert centers, and multidisciplinary cardio-obstetric and PH team care. The goal of management is therefore not to minimize risk by diagnostic labels alone, but to define the individual interaction between PH etiology, pulmonary vascular burden, RV adaptation, oxygenation, systemic disease, fetal status, and patient preference. A personalized approach that combines careful counselling, close monitoring, timely treatment escalation, and anticipatory peripartum/postpartum planning offers the best opportunity to achieve favorable maternal and fetal outcomes while acknowledging the persistent risk inherent to PH in pregnancy [1, 3–9, 30].

## Data Availability

No datasets were generated or analysed during the current study.

## Abbreviations

6MWD: Six-minute walk distance
BNP: B-type natriuretic peptide
ERS: European Respiratory Society
ESC: European Society of Cardiology
ICU: Intensive care unit
LV: Left ventricle
NT-proBNP: N-terminal pro–B-type natriuretic peptide
PAH: Pulmonary arterial hypertension
PH: Pulmonary hypertension
PVR: Pulmonary vascular resistance
RA: Right atrial
RHC: Right heart catheterization
RV: Right ventricle
SpO_2_: Oxygen saturation by pulse oximetry
SVR: Systemic vascular resistance
TR: Tricuspid regurgitation
